# Exercise and the Cortisol Awakening Response: A Systematic Review

**DOI:** 10.1186/s40798-017-0102-3

**Published:** 2017-10-10

**Authors:** Travis Anderson, Laurie Wideman

**Affiliations:** University of North Carolina at Greensboro, Greensboro, NC 27412 USA

**Keywords:** Biomarker, Stress, Athletes, Monitoring, Overtraining

## Abstract

**Background:**

The cortisol awakening response (CAR) has been used as a biomarker of stress response in a multitude of psychological investigations. While a myriad of biochemical responses have been proposed to monitor responses to exercise training, the use of CAR within the exercise and sports sciences is currently limited and is a potentially underutilized variable. Therefore, the purpose of this review was to collate studies that incorporate both exercise and CAR, in an effort to better understand (a) whether CAR is a useful marker for monitoring exercise stress and (b) how CAR may be most appropriately used in future research.

**Methods:**

A systematic review of the literature was conducted, following PRISMA guidelines. Searches were conducted using PubMed, SportDISCUS, Scopus, and PsychInfo databases, using search terms related toCAR and exercise and physical activity.

**Results:**

10,292 articles were identified in the initial search, with 32 studies included in the final analysis. No studies investigated the effects of laboratory-controlled exercise on CAR. Variable effects were observed, possibly due to inconsistencies in study design, methodology, population, and CAR analysis. The available literature suggests a threshold of exercise may be required to alter the HPA axis and affect CAR. Moreover, CAR may represent a combination of previous exercise load and upcoming stress, making current interpretation of field-based observational research challenging.

**Conclusions:**

More research is needed to fully elucidate the influence of exercise on CAR and address a number of gaps in the literature, including controlling exercise load, consistent sample collection, and CAR calculation and analysis.

## Key Points

- There is sufficient evidence for the continued investigation of CAR as a potential biomarker for exercise-related monitoring, both in athletes and the general population.
- Currently, the discrepancies observed in the literature make interpretation of the findings and future recommendations difficult.
- To confirm CAR as an appropriate biomarker for use in exercise response or overtraining monitoring, it is essential that future studies follow recommended guidelines for utilizing and reporting CAR, as discussed in this review.

## Background

Monitoring the physiological responses to exercise is critical for exercise scientists in all facets of the discipline. While numerous physiological responses (e.g., resting heart rate [1, 2], HR variability [3–5], and inflammatory markers [6]) have been investigated for their potential use as a monitoring tool of physical stress in exercise or as indicators of overreaching/overtraining, a single variable capable of acting as an indicator of exercise-induced physical stress has remained elusive. Although the search for a single marker that captures an athlete’s stress or recovery continues, the likelihood of such a marker being identified is low. Therefore, many researchers have increased interest in a composite marker of stress that may represent, in a more comprehensive manner, the degree of physical stress experienced by an athlete or exercising individual. Even so, each component of such a composite marker must be individually studied and assessed for potential inclusion in the model.

The hypothalamic-pituitary-adrenal (HPA) axis is principally controlled through corticotropin-releasing hormone (CRH) secretion from the hypothalamus. AVP (arginine vasopressin) may also act synergistically with CRH to stimulate adrenocorticotropin hormone (ACTH) synthesis and secretion from the anterior pituitary. The increased ACTH concentration then activates adrenocorticotropic receptors on the adrenal cortex to stimulate secretion of the steroid hormone cortisol. Circulating cortisol consists of primarily the bound, inactive form of the hormone, while 5–10% is unbound and biologically active and plays a prominent role in a variety of functions, including metabolic, immune responses and psychological effects through binding to cytoplasmic glucocorticoid receptors.

Due to this hormonal cascade, cortisol concentrations are controlled by the secretion and synchrony of CRH and AVP, which, during periods of low stress, are secreted in a pulsatile manner approximately 2–3 times per hour. As the hormonal end product, cortisol acts in a negative feedback manner, suppressing activity at the hippocampus, hypothalamus, and pituitary glands [7]. Cortisol shows strong diurnal variation, peaking following quiescence (i.e., shortly after waking) [8]. This diurnal pattern is controlled by a complex set of interactions initiated by the so-called biological clock in the suprachiasmatic nucleus (SCN) of the hypothalamus. In brief, there exists a self-oscillating transcriptional loop in the nucleus of SCN cells. A circadian locomotor output cycle kaput (CLOCK) and brain-muscle-arnt-like protein 1 (Bmal-1) heterodimer binds to DNA response elements to stimulate the expression of periods and cryptochromes, which phosphorylate and negatively feedback on CLOCK and Bmal-1 to prevent further gene expression [9]—a cycle which takes approximately 24 h [10]. The SCN also incorporates external feedback, such as light exposure via the optic nerves. Although the HPA axis and circadian rhythmicity are intricately linked, a complete review of the interactions between the HPA axis and the master circadian CLOCK systems are well beyond the scope of this exercise and CAR-related review. Interested readers are encouraged to consult several comprehensive reviews available on this topic (see Nadar et al. [10], Nicolaides et al. [9], and Wiley et al. [11]).

During acute stress, a dramatic increase in CRH and AVP pulsatility will ultimately increase circulating cortisol concentrations. Exercise serves as such a stressor, resulting in the aforementioned higher-order brain centers recognizing a threat to homeostasis and responding accordingly. As such, cortisol is a common biomarker that is used in the analysis of exercise responses in both elite athletes and clinical populations. Interestingly, elevations in acute and basal cortisol concentrations in response to stress are believed to be detrimental to health, while elevations in acute and basal cortisol levels in response to chronic exercise are thought to be beneficial. The mechanism related to this exercise-cortisol paradox has been speculated to be partially linked to medial prefrontal cortex dopamine levels and glucocorticoid receptors, but most of this evidence is limited to animal studies (see Chen et al. [12] for review). While it is well known that cortisol concentrations will increase in response to acute exercise, it is important to note that this occurs only when appropriate intensity thresholds have been achieved [13].

The primary function of cortisol secretion in response to exercise is to increase the availability of substrates for metabolism, both during the activity [14] and into recovery [15]. It has been shown that cortisol may then exhibit a “rebound” effect and remain depressed for 24–48 h after exhaustive exercise [16]. This disruption to the HPA axis has implicated cortisol as a potential biomarker for diagnosing overtraining, although this effort has yet to yield consistent findings [17]. Moreover, exercise training may influence acute HPA responses to exercise, decreasing pituitary sensitivity to negative feedback [15] or increasing peripheral tissue-level sensitivity to cortisol [18]. There is also significant research on the role of cortisol in obesity (see Rodriguez et al. [19] for review). Somewhat paradoxically and despite the role of cortisol as a primary lipolytic hormone, elevations in cortisol can result from an elevated body fat content (see McMurray and Hackney [20] for review), thus suggesting that changes in resting cortisol concentrations may be of interest in monitoring effectiveness of exercise and weight loss programs, especially in individuals who are obese.

In addition to the diurnal pattern of cortisol secretion, a distinct rise in cortisol has been observed immediately after waking [21], typically peaking 30–45 min after waking [22], and has been appropriately termed the cortisol awakening response (CAR). This response is a neuroendocrine manifestation of the HPA axis, considered to be superimposed over the regular diurnal cortisol rhythm [23], and has been demonstrated to be sensitive to a host of psychological conditions and stressors. CAR is believed to act as a “boosting” mechanism, to aid in physiologically preparing one for waking somatic tasks [24]. This rationale is primarily due to CAR being present independent of postural condition [22] and use of an alarm clock [25]. While light does appear to affect the response [26], the absence of optical stimuli does not seem to eliminate the response entirely. Thus, the act of waking may be considered to be an event that disrupts homeostasis [10], resulting in increased pulsatile frequency at the hypothalamus and culminating with increased cortisol secretion. The hippocampus has been touted as playing a “central role” in regulating CAR [27], while several other brain regions have been implicated in the fine-tuning of CAR, including the limbic system.

There have been a number of methods developed for assessing CAR that are worth briefly discussing. As is standard in a range of endocrine research areas, especially those that include a specific time series, researchers often calculate the area under the curve of the cortisol awakening response (AUC), typically over 1–4 measures taken after the initial awakening event. This can be calculated using two predominate AUC methods [28]: the AUC can be represented relative to a 0 concentration point (termed AUC relative to the ground [AUC_g_]) and/or AUC that reflects only the increase in concentration observed (AUC_i_). Thus, AUC_g_ represents the total hormonal exposure, while AUC_i_ is the total increase in exposure following waking.

Although it is currently unknown whether CAR and AUC_g_ or AUC_i_ reflect dissimilar physiological phenomena, these markers can show disparate responses to the same intervention. In addition to exposure measures, there are several other approaches to examining CAR, including the relative increase in cortisol, calculated as a percent increase above the first sample concentration (CAR_%_). Researchers may also calculate the mean increase (MnInc) by averaging the increased cortisol concentrations above the first sample concentration, the morning cortisol peak (CMP), or mean morning cortisol (i.e., the mean of serial morning samples in the awakening period; CAR_μ_). Also frequently assessed is the calculated slope of the CAR response, typically between the first sample and peak (CAR_slope_). Lastly, contrast effects (e.g., linear or quadratic) can be used to assess the shape, or change in shape, of CAR. As is often the case when hormones are investigated, there appears to be significant inter-individual variability in both the cortisol profile and CAR in response to physical stress. However, the CAR response within a given individual seems to be consistent [29], as long as confounding variables are properly controlled, such as time of waking, sex, and age (see Clow et al. [30] for detailed review of confounding variables).

Until recently, a vast majority of CAR research has occurred in the psychobiological literature, where CAR has been related to burnout [31], chronic fatigue, and stress [32, 33]; depression [34]; and post-traumatic stress disorder [35]. Although normative ranges have been developed for several populations [36], it is still unclear what may constitute a “healthy” CAR, as both elevated and depressed responses have been related to dysfunctional psychosocial health status [37].

These changes in CAR in relation to psychological stress raises the possibility of CAR also being an appropriate measure to monitor responses to physiological stressors (i.e., exercise). The benefits of a biomarker such as CAR are severalfold. Firstly, measures can be obtained via saliva which is less invasive to obtain as compared to biomarkers obtained from plasma or serum. Secondly, and in contrast to other assessments of overstrain which require an athlete to complete multiple exercise sessions [38], the measures can be obtained at rest, greatly reducing subject burden. Lastly, the multitude of factors that are thought to affect CAR could confer a potential marker of global stress (e.g., allostatic load), which may be more important for monitoring health or potential of burnout or overtraining in athletes. Since the symptomology of the overtraining syndrome includes psychosocial disturbances which accompany the depreciation of physiological factors, CAR may be useful in monitoring both components simultaneously.

Presently, the use of CAR in the exercise science literature has been limited and variable. Therefore, the purpose of this review was to collate the results of studies that have investigated the impact of exercise or physical activity on CAR, in an effort to understand how this biomarker could be better utilized in the exercise and sports science fields.

## Methods

A systematic review of the literature was conducted with the collection of articles concluding on November 4, 2016. The PRISMA guidelines for systematic reviews and meta-analyses were followed [39], except where not applicable. Searches were completed via the electronic search databases PubMed, SPORTDiscus, PsychINFO, and Scopus to identify publications that included markers of CAR and physical activity, exercise, and/or physical fitness. The search terms utilized were all possible combinations of terms from List 1 and List 2 (Table 1). In addition, previous review articles and relevant publications were also analyzed for any citations which may meet inclusion criteria. Due to the novelty of CAR within the exercise science literature, no restrictions in the search terms were used, such as date ranges or place of publication; however, only articles written in English were included in analysis. All searches were completed November 4–5, 2016.

**Table 1:** Search terms

List 1		List 2
Cortisol awakening response	AND	Exercise
Cortisol response to awakening		Physical activity
Awakening cortisol response		Sport
CAR		Training
ACR		Competition
CRA		Athlete
		Physical inactivity
		Physical fitness

The first screening of the articles excluded studies based on the relevance of the title of the publication, the second screening excluded studies based on a reading of the abstract, and the third screening excluded studies based on a reading of the full text. To be eligible for inclusion, studies needed to be primary, peer-reviewed research. In addition, studies must have had at least one marker of CAR and an objective measure of physical activity, exercise, or physical fitness. In the event that data was not available in the manuscript, efforts were made to contact the corresponding author to acquire the necessary data. Inclusion in the study was not limited by study design, length of intervention, or CAR methodology.

This review had two primary aims: (1) to summarize the current state of CAR in regards to exercise and physical activity and its potential use as an exercise-related biomarker and (2) to provide recommendations to researchers for future exercise and CAR studies. It is important to note that the authors originally intended this review to result in a meta-analysis of alterations in CAR in response to both acute and prolonged exercise protocols. However, due to the limited number of studies, disparate protocols, and outcome variables; range of populations studied; and variable statistical analyses used, it was determined that attempts to summarize the findings in these studies would not result in any further clarification of the influence of exercise on CAR. Therefore, no aggregated data were used in any statistical analyses, nor were any inter-study composite measures reported in the present study.

## Results

**Fig. 1:**
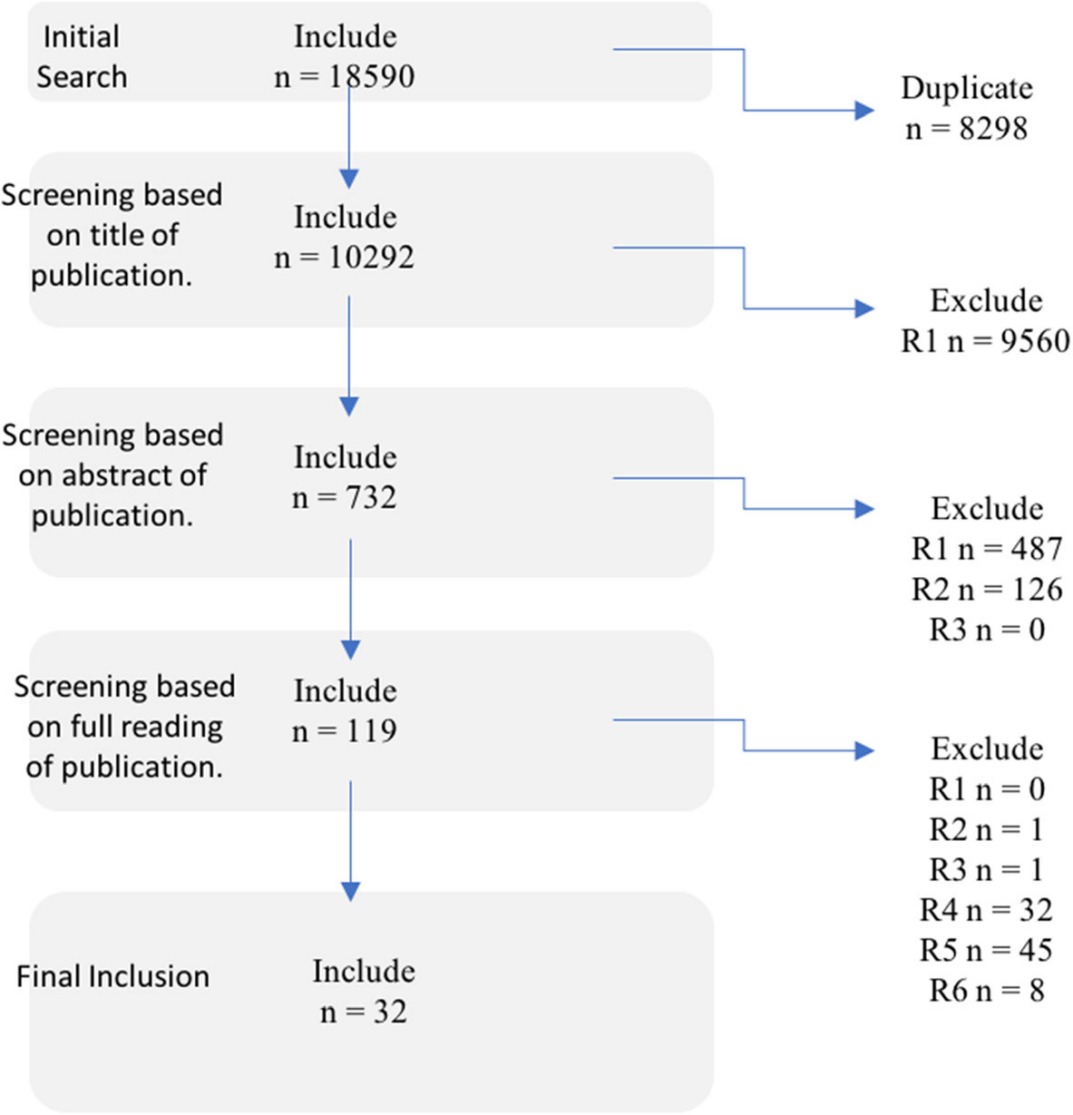
Flowchart of literature search and exclusion. Note:R1 not relevant to the topic, R2 not primary, peer reviewed source, R3 not in English, R4 no objective marker of exercise, R5 no measure of CAR, R6 duplicate

A total of 10,292 articles were identified in the initial search. Following the subsequent screenings, 32 articles were included in the review (Fig. 1).

Of the articles included, 11 articles concerned athletic populations, and a single article addressed a military population. The remaining studies included a variety of populations, including psychiatric illness (*n* = 3), children and adolescents (*n* = 5), older adults (*n* = 6), and obese populations (*n* = 2).

Articles included in this analysis showed four distinct types of exercise and physical activity measures: (a) responses to exercise intervention ≥ 1 week (Table 2, *n* = 13); (b) response to a single exercise bout (Table 3, *n* = 2); (c) relationships to physical activity (Table 4, *n* = 12); and (d) response to upcoming exercise stress (Table 5, *n* = 5).

**Table 2:** CAR as a response to prolonged exercise intervention (*n* = 13)

Citation	Population	Variable	Exercise intervention	Results
Athletes				
Filaire et al. [45]	12 adolescent female tennis players; 14.8 ±0.6 years	Saliva; 0 h, + 0.5 h; CAR, AUC_g_	16 weeks, tennis training program	↓CAR, ↓AUC_g_
Gouarne et al. [42]	9 untrained males (25.1 ± 1.4 years), 10 male triathletes (26.5 ± 2.7 years)	Saliva; 0 h, + 0.5 h; CAR_%_	10 months, triathlon training	↑CAR_%_ in OT athlete
Gunnarsson et al. [41]	12 crew members of Volvo Ocean Race; 32 years	Saliva; 0 h, + 0.3 h, + 0.6 h; CAR	9 months, round-the-world sailing competition	↓CAR
Minetto et al. [40]	15 male soccer players; 24.4 ± 3.9 years	Saliva; 0 h, + 0.25 h, + 0.5 h; AUC_g_, slope	7 days intensified soccer training (60% greater than preceeding months)	↑0 h, AUC_g_,
Park et al. [43]	25 male trekkers (14–59 years), 21 male Sherpas (16–39 years)	Saliva; 0 h, + 0.5 h, + 1 h; CAR, AUC_g_	Trek to 4800 m	↑CAR, AUC_g_@4800 m in trekkers
Military				
Clow et al. [49]	13 male, 7 female military recruits; 17–24 years	Saliva; 0 h, + 0.25 h, + 0.5 h; CAR, AUC_g_, MnInc	11 weeks, basic military training	↓AUC_g_ at week 3 and 6
Non-athletes—yoga				
Curtis et al. [50]	22 female; fibromyalgia; 47.4 ± 13.7 years	Saliva; 0 h, + 0.5 h; CAR	Hatha yoga; 2 × 75-min classes, 8 weeks	No change
Daubenmier et al. [51]	Female; BMI 25–40 kg m^−2^, < 300 lbs	Saliva; 0 h, + 0.5 h; CAR, slope	9 × 2.5 h + one 7-h guided meditation (inc. yoga)	No change
Non-athletes—aerobic exercise				
Calogiuri et al. [56]	7 male, 7 female sedentary-mod. Active; 49 ± 8 years	Saliva; 0 h, + 0.25 h, + 0.5 h; AUC_i_, AUC_g_	25 min cycling, 20 min resistance; mod-high intensity (RPE), indoor and nature groups	AUC_i_: indoor > nature (*p* = 0.04)
Foley et al. [52]	23 subjects, experiencing major depressive episode or taking antidepressant	Saliva; 0 h, + 0.5 h; CAR	12 weeks, 30–40 min mod. Aerobic exercise	↓CAR @ 6 and 12 weeks
Foss et al. [53]	15 subjects; 45.2 ± 9.6 years; BMI > 35	Saliva; 0 h, + 0.5 h; CAR	22 weeks, 3 days/week, group-based mod.-vig. exercise	No change
Menke et al. [55]	12 males w/burnout; 12 male controls; 45.4 ± 6.2 years	Saliva; 0 h, + 0.17 h, + 0.34 h, + 0.5 h; AUC_g_	12 weeks, 2–3 days/week, aerobic exercise@60–75%HR_max_	No change
Tortosa-Martinez et al. [54]	21 persons diagnosed with amnestic mild cognitive impairment; 75.5 ± 7.23 years	Saliva; 0 h, + 0.5 h; CAR	Group exercise; 3 months, 3 days/wk., 60 min aerobic exercise@60–75%HR_max_	No change

*BMI* body mass index, *RPE* rating of perceived exertion, *HR*_*max*_ maximum heart rate, *OT* overtrained

**Table 3:** CAR as a response to a single exercise bout (*n* = 2)

Citation	Population	Variable	Exercise intervention	Results
Garde et al. [57]	21 regular exercisers; 43.0 ± 11.6 years	Saliva; 0 h, + 0.5 h; CAR	1.5 h gym session inc. resistance/circuit training	No change
Ucar et al. [58]	20 male medical students 20–24 years	Saliva; 0 h, + 0.25 h, + 0.5 h, + 1 h; AUC_g_, AUC_i_	90 min soccer match	No change

**Table 4:** CAR relationships to physical activity (*n* = 12)

Citation	Population	Variable	Physical activity measure	Results
Physical activity monitoring				
Bogg & Slatcher [59]	960 adults; 25–74 years	Saliva; 0 h, + 0.5 h; CAR	Physical activity survey, vig./mod., general activity	No relationships
Dubose & McKune [66]	23 females children; 8.4 ± 0.9 years	Saliva; 0 h, + 0.5 h; AUC_g_	5-day tracking via Actigraph	Vig. and MVPA vs. + 0.5 h (*r* = .52, .39), vig vs. AUC_g_ (*r* = .46)
Eek et al. [60]	352 female, 229 male; 46.3 ± 10.7 years	Saliva; 0 h, + 0.5 h; CAR_%_, CAR_μ_, CMP	SOFI-20	CAR vs. LoE (*r* = .11), PE (*r* = .11); 0 h vs. PE (*r* = −.1)
Heaney et al. (2014) [72]	36 community-dwelling adults; 65–88 years	Saliva; 0 h, + 0.5 h; CAR	West of Scotland Twenty-07 Study	No difference based on activity level
Heaney et al. (2012) [86]	24 students (20 ± 1.16 years) and 48 older adults (75.6 ± 6.35 years)	Saliva; 0 h, + 0.5 h; CAR	Whitehall Study questionnaire	No relationships
Martikainen et al. [64]	272 adolescents; 12.4 ± 0.5	Saliva; 0 h, + 0.25 h, + 0.5 h, + 0.75 h, + 1 h; AUC_g_	12 h Actiwatch; PA, ST, vig. PA	Female AUC_g_ partial correlations with PA, ST, vig. PA
McHale et al. [69]	28 adolescents; 13.3 ± 2.3 years	Saliva; 0 h, + 0.5 h; CAR, peak	DISE-YV	No effects
Pulopulos et al. [68]	86 older adults; 64.42 ± 3.93 years	Saliva; 0 h, + 0.5 h, + 0.45 h; AUC_i_	PA, walking speed	AUCi correlated with WS (*r* = −.223); PA significant covariate in regression model
Strahler et al. (2010a) [71]	Older and younger ballroom dancers and controls	Saliva; 0 h, + 0.5 h; CAR, AUC_g_	PA (h/week)	No relationship
Vreeburg et al. [61]	418 adults from Netherlands Study of Depression and Anxiety; 43.0 ± 14.7 years	Saliva; 0 h, + 0.5 h, + 0.45 h, + 1 h; AUC_g_, AUC_i_	Physical Activity Questionnaire	Partial correlations with PA and AUC_g_ (β=.13) AUC_i_ (*β*= − .11)
Zeiders et al. [70]	100 adolescents of Mexican-origin families; 15.3 ± 0.5 years	Saliva; 0 h, + 0.5 h; CAR	Daily exercise self-report	No relationship
Physical Function Tests				
Gardner et al. [73]	962 males; 73.4 ± 4.17 years; CaPS cohort	Saliva; 0 h, + 0.5 h; CAR	“Get up and go” and “flamingo” tests	No relationship

*CaPS* Caerphilly Prospective Study, *vig* vigerous*, mod* moderate, *SOFI-20* Swedish Occupational Fatigue Inventory, *PA* physical activity, *WS* walking speed, *ST* sedentary time, *PE* physical exertion, *LoE* lack of energy

**Table 5:** CAR response to upcoming exercise stress (*n* = 5)

Citation	Population	Variable	Exercise intervention	Results
Balthazar et al. [75]	8 male triathletes; 27.8 ± 3.2 years	Saliva; 0 h, + 0.5 h	2–4 h triathlon (1.5 km swim, 42 km cycling, 10 km run) vs. rest day	No diff. in CAR, ↑0 h and ↑+ 0.5 h on Comp day
Diaz et al. [77]	11 male swimmers; 21.5 ± 2.16 years	Saliva; 0 h, + 0.5 h, + 1 h; CAR, AUC_g_, AUG_i_	Major swimming competition	No difference (C vs. Comp).
Labsy et al. [74]	9 male soccer players; 19.9 ± 0.4 years	Saliva; 0 h, + 0.5 h; CAR	Morning/afternoon exercise 45 min@70%HR_peak_ + 2 × 15min@80%HR_peak_ + 5 × 1 min@HR_max_	No relationship
Meggs et al. [78]	41 competitive swimmers; 15.2 years	Saliva; 0 h, + 0.25 h, + 0.5 h; AUC_g_	100 m swimming competition	AUC_g_ relationship with performance (*β* = .321)
Strahler et al. (2010b) [76]	12 national martial arts athletes	Saliva; 0 h, + 0.25 h, + 0.5 h, + 0.75 h, + 1 h; AUC_g_	National martial arts competition	No relationships

*HR*_*peak*_ heart rate peak, *C* control, *Comp* competition

### Response to a Prolonged Exercise Intervention

Studies observing prolonged exercise interventions included a number of populations and exercise interventions. In an attempt to more clearly understand the responses observed, we discuss the impact on athletes and non-athletes separately.

#### Athletes

In the five studies identified as monitoring the response of the CAR to an exercise intervention in athletes, the variables of interest included CAR, CAR_%_, and AUC_g_, and the interventions ranged from a 7-day intensive soccer training period [40] to a 9-month global boat race [41]. Clearly, the variability in the populations assessed, the techniques for assessing the awakening response, and the statistical techniques used make the aggregation of the data nonsensical. However, some patterns did become evident. For example, Gouarne et al. [42], Minetto et al. [40], and Park et al. [43], all showed an increase in CAR following training (triathlon, soccer, and mountain climbing, respectively). More specifically, Gouarne et al. [42] demonstrated that elite-level triathletes will exhibit an increase in CAR_%_ following the onset of training but will stabilize at a higher level even as the season progresses, which appears to mirror the findings in mountain climbers [43]. It is important to note here that although Park et al. framed this particular study in terms of altitude exposure, it is reasonable to include mountain climbing as a form of prolonged exercise. Even so, the interpretation of the results of this study is more difficult due to the additional impact of altitude on cortisol responses [44]. Nonetheless, there was a clear increase in the CAR of mountain climbers after climbing to 4800 m in 8 days, compared to only a single day of climbing to 1100 m. The lack of a similar response by Sherpas to the same intervention was suggested by the authors to be a result of acclimatization to the altitude, although this may also be viewed as the response of a well-trained and adapted individual for this specific exercise stress. Thus, one could interpret the study as being the same exercise load applied to well-trained and lesser-trained individuals, with the latter group showing larger increases in CAR. Again, this suggested initial increase in CAR could be expected following a significant exercise intervention, which may then level off as participants become accustomed to the exercise load.

These findings seem contrary to Filaire et al. [45], who demonstrated that a 4-month tennis training period in players aged 14.8 years with an average of 7+ years of training, resulted in a decrease in AUC_g_ and CAR. As a potential explanation of this discrepancy, it was reported that subjects also exhibited a disturbance in REST-Q subscales indicating a decreased affective state; a significant relationship between CAR and these psychological affects was present. These findings suggest that CAR will be altered differentially, contingent on the physiologic response of the athlete to the training load imposed on them. That is, if the training load is too great for an individual’s fitness level, they may present an inverse response (decreases in CAR), relative to another athlete who responds to the same training load in the opposing direction (increases in CAR). In support of this rationalization, Gouarne et al. [42] observed that two triathletes developed the overtraining syndrome across the course of the triathlon season, as determined both by a decrease in athletic performance, as well as decreases in subjective fatigue scores [46]. In those overtrained athletes, and juxtaposed with the non-overtrained athletes, CAR_%_ showed a decline in response to the training, eventually stabilizing in one of the athletes, but not the other. Although it is difficult to compare the results directly, a global boat race appears to have blunted the awakening response as the race progressed over 9 months [41]. The authors likened this response of the sailors to that observed in burnout patients, whom have been shown to also present a blunted CAR [31].

In contrast, and challenging the idea of blunted CAR to elevated training load in athletes, Minetto et al. [40] showed an increase in CAR and AUC_g_ in response to training, while showing a positive relationship between these responses and performance. It is possible however that due to the short training period employed in this study (7 days), these athletes were exhibiting only local muscular fatigue that affected their physical performance, but that did not reach the threshold required to negatively affect neuroendocrine function during awakening.

In summary, these studies posit the possibility that shifts in CAR may not be consistent across all training loads, potentially increasing as training is first imposed and stabilizing after an acclimatization period, before decreasing if training load becomes too extreme and overloads the athlete. Of course, these are speculative claims and must be investigated directly by designing longitudinal studies that investigate this specific response. Moreover, as has been done previously, intensity or load thresholds should be investigated relative to disruption of the HPA axis and a subsequent effect on the CAR in athletes. This potential non-linear relationship with training load may be reflective of a similar phenomenon observed in the chronic fatigue and burnout literature, which suggest CAR may increase [47], decrease [31], or be unchanged [48].

#### Military

Although the separation of athletic and military physical interventions could be considered arbitrary, we believed that some military personal may have had very little exposure to physical conditioning prior to their enrollment in military service, a belief that was reflected in the high proportion of subject “dropout” [49]. This lack of extended or even lifelong exposure to physical training may result in disparate CAR responses relative to athletes and is therefore discussed independently.

In the singular study that examined military physical training on CAR, Clow et al. [49] tracked military recruits across an 11-week basic training program, involving significant physical training components. The researchers used a cortisol awakening response variable termed the mean increase (MnInc), calculated as the difference of the average of the second and third sample above the first sample (MnInc = ((*s*_2_ + *s*_3_)/2) − *s*_1_). The authors found no change in MnInc, but AUC_g_ decreased significantly at weeks 3 and 6, before returning to baseline levels by week 11. Interestingly, the authors also analyzed the shape of the curves across the time period, showing linear increases at weeks 0 and 3, compared to quadratic contrasts at week 0 and week 12, again suggesting a leveling off after the initial cortisol increase. Evidently, the observed function and degree of change over time should be considered in analyses. This observation has implications for the desired sampling frequency and methodology (i.e., at least 3 samples to test for quadratic relationships, 4 for cubic relationships, and so forth).

#### Non-athletes

In studies of exercise interventions in non-athletic or untrained populations, exercise paradigms used tended to be either aerobic (i.e., cycling) or low-impact practices such as yoga. Since these intervention types likely affect the HPA axis dissimilarly, the results of these studies are discussed separately.

##### Yoga Interventions

In the two studies observing changes in CAR in response to yoga practice, populations studied included those patients diagnosed with fibromyalgia [50] and a non-clinical sample of female subjects [51]. Inconsistent findings are therefore somewhat expected given that CAR has been shown to be altered in clinical populations, likely because these individuals are often under severe chronic physical, emotional, financial, and psychological stress. Cutis et al. [50] studied changes in CAR following 8 weeks of yoga training in a clinical fibromyalgia population. The area under the cortisol curve was reported to significantly increase following yoga training; however, the authors included an evening cortisol sample in this AUC calculation, as opposed to analyzing only the awakening response. Even so, the data presented shows that the area was mostly influenced by the first two measures (i.e., the CAR). Therefore, the yoga program appears to have increased AUC, with no change in the slope of the response. In a non-clinical population, Daubenmier et al. [51] observed no change in CAR following a 9-week mindfulness intervention, which included a component of yoga training, relative to a control group. The details of this program were significantly lacking, so it is difficult to interpret this result relative to those of other studies.

From the limited number of studies, it is likely that low-intensity, low-impact physical activity has little impact on CAR, unless the subject is under severe chronic stress from a clinical condition. In this case, low-intensity exercise may have a positive effect on CAR moving it toward a “normal healthy” response and away from either an over-responsive or under-responsive CAR. More studies are required to fully elucidate these effects, in both clinical and non-clinical populations.

##### Aerobic Exercise

The impact of aerobic exercise on CAR in non-athletes was assessed in five studies. In four of the five studies, no statistically significant change was reported, although the details of these investigations are interesting to consider. For example, Foley et al. [52] showed aerobic training non-significantly decreased CAR after 6 and 12 weeks. The decrease observed at week 12 (week 12 1.89 ± 1.30 ng/ml vs. week 0 3.59 ± 2.63 ng/ml) certainly implies a decrease in CAR, but the results are impeded by a small sample size (*n* = 8) and large inter-individual variability. Similarly, in a sample of subjects presenting a BMI > 35 (i.e., obese), a lifestyle intervention (including an aerobic exercise program) resulted in morning cortisol being greater than that observed in matched controls at both 0 h and + 0.5 h, although neither the treatment nor control group showed a significant change from their respective pre-intervention values [53]. In this instance, it is likely that utilizing AUC (or associated AUC-related variables) may have conferred more information than simple cortisol values taken at individual time points. Interestingly, the morning increase was greater in the intervention group at 6 months of follow-up compared to the control group, suggesting there are residual effects on CAR following exercise interventions, although it is unclear why this would not also be observed immediately following the exercise intervention. Indeed, the lack of details of subject activities during this period makes any inferences to this point problematic. Comparably, Tortosa-Martinez et al. [54] showed no statistical difference between control and intervention groups in CAR post-intervention, although an increased peak at +0.5 h and the magnitude of the increase was nearly significant (*p* = .068).

Finally, in a study in participants suffering from job-related exhaustion (and therefore a suspected impaired CAR), there did not appear to be any change in CAR following the 12-week aerobic exercise intervention [55]. As with the clinical populations discussed above, in those populations that likely have a chronic shift in their CAR, any additional or consistent alteration in CAR seems doubtful. This null finding may in fact be considered a positive effect of exercise in these chronically fatigued individuals. It is possible that exercise prevented any further shift in CAR in these subjects (i.e., perhaps a basement effect was observed). Of course, longitudinal analysis of this population should be conducted to test these hypotheses.

In the single study that observed a change in the CAR, a “green-exercise” intervention in which office workers were prescribed exercise either in an indoor or an outdoor setting was investigated [56]. Figures included in this study suggest that both indoor and outdoor exercise changed the shape of the CAR. That is, the indoor group moved from a “regular” CAR, with a peak occurring at + 0.25 h, to a decreased cortisol concentration at the same time following the intervention. The outdoor group on the other hand showed only a small increase at + 0.25 h pre-intervention but moved to a more regular response post-intervention. In this case, reporting linear or quadratic contrasts may have been beneficial in fully explaining the shift in CAR. Moreover, the outdoor exercise appeared to have a much smaller AUC_i_ than the indoor exercise following the intervention. This was, however, likely due to the relative decrease at + 0.25 and + 0.5 h post-awakening cortisol observed in the outdoor group following exercise. This inverted awakening response is indeed curious but may be an artifact of the small sample size (*n* = 6) and the inherent variability in cortisol responses.

### Response to a Single Exercise Session Intervention

Compared to more prolonged interventions, two studies observed possible changes in CAR following a single exercise bout. While small in number, these investigations are crucial if CAR is to be considered as an appropriate measure for monitoring day-to-day responses to exercise interventions.

In one of the studies observing CAR response to a single exercise session, the researchers found no differences in the awakening response the morning after an exercise bout, consisting of a resistance exercise, circuit-training-type exercise protocol [57]. It must be noted that the circuit training was approximately 20 min, followed by a 15-min period of “fitness training.” No reference was made to what this may have included, but it was presumably some type of aerobic training. Therefore, approximately 35 min of physical exercise was completed, with no reference to the intensity or participant exertion during the session. Previous literature on the acute exercise-induced response of cortisol indicates that an intensity threshold exists [13]. Thus, if we are to understand CAR to reflect the cumulative stress over time, a relatively short bout of exercise that is not particularly intense may not produce any alteration in CAR, since it is unlikely to illicit acute HPA responses.

Similarly, in response to a late-night exercise bout, Ucar et al. [58] observed CAR responses to a soccer match in college-aged participants. The serial sampling protocol showed no differences in any of the morning time points following an evening soccer match compared to a control condition, although it appears as if the initial peak may have been greater following the match, thus making the AUC_i_ variable non-significantly greater. Again, the variability in these responses (1 h AUC_i_ range 439–70,003 a.u.) makes analyses difficult, and therefore, intra-individual responses, even within a given population, may be more beneficial.

### Response to Daily Activity Monitoring

Studies observing the CAR in relation to physical activity were numerous; however, few directly assessed the impact on CAR and instead included physical activity as a component in a multivariate model. For example, Bogg and Slatcher [59] assessed the impact of physical activity on CAR via multi-level growth curves, thereby making extracting data and possible relationships difficult. Nonetheless, in this particular study, no significant intercepts were observed for general physical activity in any of the models. Of note, moderate-vigorous activity approached a significant correlation (*p* = 0.088), although such an effect would have been small and perhaps trivial.

Similarly, another study found a significant relationship with Swedish Occupational Fatigue Inventory subscales [60]. CAR was positively related with a lack of energy (*r* = .11), lack of motivation (*r* = .10), and lack of physical exertion (*r* = .11). Noticeably, these are only weak or very weak relationships, likely observed to be statistically significant because of the large sample size (*n* = 581) included in the analysis. Nonetheless, and quite interestingly, when analyzed by sex, these relationships held only in females and no significant relationships were observed in males. Even though there were fewer males in this study, the authors state definitively that the lack of relationships in males were not due to lack of statistical power; rather, there may be a true difference in responses between males and females. It must be noted however that this study did not control for any objective marker of exercise or physical activity, and thus the impact of non-work related physical activity remains unclear.

A study by Vreeburg et al. [61] showed significant relationships between AUC_g_ and AUC_i_ and physical activity in depressive subjects. Interestingly, these relationships were inverted relative to each other, such that physical activity was a positive predictor of AUC_g_ and a negative predictor of AUC_i_, suggesting an overall greater magnitude of cortisol output, yet a blunted cortisol response in those individuals who are more active. Since depressive patients have been shown to have blunted responses (i.e., lowered AUC_i_) relative to healthy controls [62, 63], this finding may indicate that physical activity was beneficial in moving the CAR to a more healthy profile.

Also assessing physical activity via multivariate models, Martikainen et al. [64] found partial correlations between the awakening AUC and overall and vigorous physical activity. However, the modeling techniques also adjusted for a number of variables, including the timing of a dexamethasone suppression test. The results are therefore difficult to interpret and generalize; however, there does seem to be evidence present that AUC_g_ shows a negative relationship with both vigorous and overall physical activity. Even though 272 adolescents were included in the study, these significant models were observed only in girls, as observed by Eek et al. [60]. It follows then that not only should sex be considered when assessing CAR in adolescents but also pubertal development. Furthermore, Ozgocer and colleagues recently showed CAR to be affected by menstrual cycle phase [65] and should therefore be controlled for in future CAR research designs.

Compared to survey-based assessments of physical activity and exercise, DuBose and McKune [66] used Actigraph technology to monitor the activity levels in 23 children and demonstrated a relationship between AUC_g_ and vigorous activity. This suggests those children who participate in a greater amount of intense physical activity also tend to show a greater awakening response. This of course must be understood in the context of a correlational analysis, as there are numerous factors that could contribute to the difference in activity level and cortisol response, especially in an adolescent population [67]. Interestingly, while there was no difference in the cortisol concentration immediately after waking, there was a relationship between the 0.5 h cortisol and both vigorous and moderate-to-vigorous activity. These findings do indicate that CAR represents a separate construct than a simple measure of basal cortisol level. In contrast, AUC_i_ was found to be significantly negatively related to walking speed (*r* = − 0.223) in older adults [68], suggesting a lower cortisol response is related to greater physical function. In addition, AUC_i_ contributed an additional 5% to the second step of a regression model predicting walking speed from a number of demographic- and health-related variables [68].

In comparison to these findings, other studies found no relationships between CAR and physical activity in adolescents in sporting clubs [69], an ethnic minority group [70], or young or old dancers [71] and no CAR differences in older adults who completed 1 or more hours per week of physical activity compared to those who did not [72].

#### Physical Tests

In addition to those studies assessing the relationship between CAR and physical activity, one study was included that measured physical and functional ability. In this investigation, Gardner et al. [73] studied 962 middle-aged and older men. No relationship was found between CAR and the “get up and go” or “flamingo” functional tasks; although after adjusting for other covariates and similarly to Pulopulos et al. [68], there was a weak relationship with CAR and walking speed only in those subjects identified in the highest CAR. AUC did not elucidate anything any further in this study.

### Upcoming Exercise Stress Intervention

Lastly, five studies assessed changes in CAR as a response to an upcoming exercise stress. This anticipated stress reaction is considered to be psychological in nature, but it is nonetheless important to consider that any CAR variable is a result of both psychological reactivity to upcoming events and previous exercise training or stress.

Three studies found no change in CAR in response to an upcoming laboratory exercise session [74], triathlon event [75], or martial arts competition [76]. However, Balthazar et al. [75] found that there was a significant increase in both awakening and + 0.5 h cortisol, but this was not accompanied by a change in CAR. The authors noted that this lack of difference was due to the large variability in the awakening response on the morning of the competition. This suggests that some athletes were perhaps more psychologically aroused by the upcoming competition than others. Similarly, since Labsy et al. [74] utilized a laboratory-based exercise session with little competitive incentive, it is likely that the psychological impact was rather low. As an alternate explanation, Strahler et al. [76] suggests that their lack of significant findings may be due to some degree of endocrine habituation in high-level athletes. In comparison, a study in competitive swimmers demonstrated a significantly greater AUC_g_ on competition days compared to control days [77]. In further support of the psychological impact on CAR, these researchers collected data on 2 days prior to the event and showed that the response appears to be much greater on day 2, which was closer to the time of competition and presumably a time of greater psychological stress.

Also evaluating swimmers, Meggs et al. [78] showed that AUC_g_ contributed significantly to a linear regression model predicting athletic performance in a swimming competition. There was also an interaction between psychological resilience, as measured by the Academic Resilience Scale [79] and AUC_g_ with performance, with the greatest performance occurring in those with low AUC_g_ and high psychological resilience. This implies that CAR may actually be beneficial in monitoring overall stress load in the athletes and may be predictive of performance in competitive events.

## Discussion

The aims of this systematic review were to firstly summarize the alterations in CAR in response to exercise and determine whether there was sufficient support for its use as an exercise-related biomarker and secondly to provide future recommendations, where warranted, for improving CAR research. We acknowledge that many of the studies included in this review include confounding variables that may influence the cortisol response above and beyond exercise alone (i.e., hypoxia due to altitude or psychological stress). However, had we limited our review to studies that investigated only the influence of exercise on alterations in CAR without any confounding variables, the number of studies included in the systematic review would have been zero. The original intent of this review was to conduct a meta-analysis and establish potential effect sizes across exercise contexts. However, as has been discussed, there are significant variations in research methodology, data reduction, populations, and statistics. Therefore, any meta-analyses presented would be inconclusive, if not entirely erroneous. As such, we aim to discuss the current state of CAR in exercise research and provide recommendations for augmenting our current knowledge of this biomarker.

From the findings presented above, it is reasonable to suggest that a threshold of exercise must be surpassed to illicit and measure a response in the CAR. Although available evidence suggests this may be intensity-dependent, future research should confirm this. The necessity of an exercise intensity threshold is not unheard of in the exercise endocrinology literature, especially in reference to cortisol [13]. The lack of change in CAR in those studies focused on low-intensity exercise or workplace stress, therefore, may in fact represent a sub-threshold effect. If true, the lack of sensitivity to low-impact exercise may be considered a potential strength of CAR, especially when considering the utility of CAR to monitor exercise training stress and thereby modulate training intensity. For example, during periods of regular exercise training, CAR may present very little variability, but overload periods of increased intensity or volume may impair the CAR, thus acting as an early predictor of overtraining.

Studies that included CAR as a physiological marker of readiness on the morning of a competition showed a degree of consistency in CAR predicting performance outcomes, leading to the suggestion that CAR potentially acts as an indicator of the psychological status of the athlete. This more positive affective state then presumably leads to improved athletic performances. However, it is also possible that the observed CAR was a function of, or affected by, the preceding chronic training load undertaken by the athletes. That is, athletes with a greater chronic training load also had more favorable physical adaptations that lead to improved performances, and that greater training load was also reflected in the CAR. Being unable to distinguish this point, there is a clear need for laboratory-controlled exercise programs that also monitor CAR.

Many studies in the present review were excluded due to their lack of a CAR measure. Of these, a significant number stated that awakening responses were collected; however, these measures were either not reported (i.e., only included concentration values at individual time points) or were reported as a composition score of “diurnal cortisol,” which included a number of cortisol measures outside of the waking period. Although there are certainly uses for monitoring the entire diurnal period, it is recommended that the awakening-period cortisol be analyzed as a separate variable, as described below, in addition to the diurnal rhythm. This small addition to the calculations and analysis may lead to relationships or changes that have been otherwise unidentified.

### Future Directions

A clear issue in the literature is related to the most appropriate way to present CAR variables. It is evident that CAR and AUC measures may both be valuable for evaluating responses to exercise, yet it remains unclear whether AUC_i_ or AUC_g_ should be more consistently used. Even more concerning is the lack of agreement in the number or timing of sample collections. While most studies include samples immediately after waking and a second sample after 30 min, the variability in studies including a third, fourth, or fifth measure, or researchers that prefer a 20- and 40-min capture period, leads to difficulties in assessing overall effect sizes. After reviewing the literature, we suggest sampling at least every 15 min following an initial waking sample, for at least 1 h after waking. This allows for the following: (1) an increased confidence in capturing the peak change; (2) an increased resolution in the shape of the response; (3) more meaningful statements in regards to total cortisol exposure (i.e., area under the curve); (4) more sophisticated statistical analyses to be completed, such as non-linear metrics; and (5) the potential to elucidate intra-individual variability in the measure across the course of an exercise training or intervention period. Of particular importance, a recent study by Smyth et al. [80] suggests there is a non-linear rise over the initial waking period, which is subsequently followed by a more linear increase, again emphasizing the need for frequent sampling.

Moreover, we recommend the reporting of at least: CAR, CAR_%_, AUC_g_, and AUC_i_, and contrast analysis. These variables allow for analysis of both the relative and absolute increases and exposure observed, as well as general shapes of the CAR curve. Since research on CAR within the exercise literature is still in its infancy, the reporting of these variables, even if not significant will (1) more fully explain the response to the intervention and (2) allow researchers to more efficiently focus on CAR variables which may be most relevant moving forward.

Field-based and observational studies are undoubtedly important for establishing the effect of exercise and CAR in real-world scenarios and outlining potential uses for this biomarker, although there is currently a lack of control over the exercise interventions, as well as potential confounding factors in the literature. As such, although CAR has routinely been obtained via saliva samples produced in the subject’s regular sleeping environment, researchers should also consider conducting studies in which subjects use sleep-in facilities so that saliva collection procedures could be more closely monitored for adherence. Although self-reports of wake-time, or activity and sleep monitors may provide evidence of waking, they do not denote the actual timing of saliva samples post-waking. As demonstrated above, the cubic or quadratic contrasts are highly time-dependent, and short delays in sampling have been shown to impact CAR [81].The use of a sleep-in laboratory facility may also allow for serum collection in conjunction with salivary measures. Previous research on salivary and serum cortisol markers have shown consistent reliability, with correlation coefficients ranging from *r* = 0.71 to 0.96 [82], depending on the population being analyzed. It is important to recognize the salivary cortisol represents only the free concentration of the hormone, and the increased activity of 11β-hydroxysteroid [83] in saliva leads to lower cortisol levels in saliva. Due to this, as well as the potential delays in free cortisol excretion through salivary glands, serum monitoring may prove to provide a more complete picture of cortisol responses during awakening. Moreover, cortisol concentrations prior to the conscious awakening point may elucidate further physiological mechanisms underlying CAR, which would be ostensibly achievable only through IV catheterization.

There are occasional references in the literature to the possibility of responders and non-responders in regards to CAR [36, 84]. It is the opinion of the authors that the lack of an increase in cortisol following awakening or the decrease in cortisol with wakening should not be excluded from analysis on the basis of being a “non-responder.” This variability in the response is critical to further understanding its nature. In the event of individual responses that do not follow the expected cortisol rise, these subjects should instead be identified and individually discussed and perhaps included in a secondary post hoc analysis.

As discussed elsewhere [45], CAR should be analyzed in regards to other possible awakening responses. These may include either the alpha-amylase or dihydroepiandrosterone awakening responses, as well as other inter-dependent physiological systems such as heart rate variability. It is likely that the relationships between these markers, as opposed to any individual biomarker, will permit more complete information regarding the response to exercise. In addition to relating CAR with other biomarkers, one should also be cognizant of the relationships that the CAR has already demonstrated with subjective markers of stress and recovery. Specifically within the exercise sciences, CAR has been studied in reference to the Profile of Mood States [77, 85], Visual Analogue Scale to Measure Fatigue [49], and an adapted Academic Resilience Scale [78]. Given the clear link between psychological stress and hypothalamic function, the inclusion of these questionnaire-based measures and monitoring of the affective state of the athlete in response to exercise training is necessary.

## Conclusions

The use of CAR in the current exercise and physical activity literature is sporadic and inconsistent. However, from the limited evidence presented, CAR appears to be a viable biomarker to monitor both exercise training responses and health-related outcomes. In particular, it appears that CAR may be influenced by an intensity threshold, since changes in CAR seem to occur in higher load interventions or those subjects which presumably have a reduced training tolerance. Moreover, CAR appears to represent physical activity in some populations and may be useful in monitoring physiology in large scale physical activity observational research. Future research should focus on addressing the methodological inconsistencies discussed above, establishing a potential exercise threshold required to illicit an acute response and determining the extent to which CAR represents past physiological disruption and upcoming exercise stress.

## Abbreviations

+*x*h: Cortisol concentration *x* hours after waking
0 h: Cortisol concentration immediately after waking
AUC_g_: The area under the curve relative to a 0 cortisol concentration
AUC_i_: The area under the curve relative to the increase in cortisol, typically immediately after waking
AVP: Arginine vasopressin
CAR: The absolute increase in cortisol from immediately after waking to peak
CAR_%_: The relative increase in cortisol from immediately after waking to peak
CAR_μ_: The mean increase in cortisol, typically taken as an average of two measures following the initial measure
CMP: The peak cortisol concentration following awakening
CRH: Corticotropin-releasing hormone
MnInc: The average cortisol concentration greater than the initial value (MnInc = ((s2 + s3)/2) − s1)
SCN: Suprachiasmatic nucleus
